# Prevalence of Sarcopenia and Frailty in Geriatric Patients with Type 2 Diabetes Mellitus

**DOI:** 10.5041/RMMJ.10554

**Published:** 2025-10-31

**Authors:** Amtoj Singh Lamba, Monica Gupta, Sarabmeet Singh Lehl, Anita S. Malhotra, Uday Pratap Singh Parmar

**Affiliations:** 1 Department of General Medicine, Government Medical College and Hospital, Chandigarh, India; 2 Department of Physiology, Government Medical College and Hospital, Chandigarh, India; 3 Government Medical College and Hospital, Chandigarh, India

**Keywords:** Diabetes, frailty, geriatrics, sarcopenia

## Abstract

**Background:**

Sarcopenia and frailty are multi-factorial conditions, but few studies have examined their prevalence among older adults with diabetes in the Indian subcontinent. This study aimed to estimate prevalence of sarcopenia and frailty in ambulatory patients ≥65 years with type 2 diabetes mellitus (T2DM).

**Methods:**

Sarcopenia was assessed utilizing the Asian Working Group for Sarcopenia (AWGS) 2019 criteria. Frailty was assessed using the Fried Frailty phenotype criteria. The study enrolled ambulatory participants aged 65 years and above with T2DM visiting the outpatient clinic. Patients with degenerative or inflammatory arthritis of the lower limbs, disabling cerebrovascular accidents, Alzheimer’s disease or other cognitive impairment, as well as those with chronic obstructive pulmonary disease, chronic liver disease, or chronic kidney disease were excluded from the study.

**Results:**

Among the 100 outpatients meeting the inclusion criteria, sarcopenia was present in 30% (including 17% with severe sarcopenia). Frailty was present in 27%, pre-frailty in 59%, and 14% were classified as robust.

**Conclusion:**

This study demonstrated a high prevalence of both sarcopenia and frailty among older adults with T2DM. Routine screening for these conditions may facilitate early identification and intervention in this high-risk population.

## INTRODUCTION

Population aging is a global phenomenon. In 2019, the global population of elderly people (aged ≥65 years) stood at 703 million; by 2050, it is predicted to double. In Eastern and Southeastern Asia, the geriatric population has almost doubled from 6% in 1990 to 11% in 2019.^1^ India is no exception to these population dynamics. As of 2021, there were over 138 million older adults in India, making up 10.1% of the country’s population, a proportion that is further expected to increase to 13.1% by 2031.^2^

Aging is accompanied by common shifts in body-composition, including reduced muscle mass and increased fat mass. Sarcopenia is defined in the literature as an age-related loss of skeletal muscle mass accompanied by a decline in muscle function assessed through measures of muscle strength, and physical performance. This condition can lead to diminished physical abilities, decreased quality of life, increased risk of falls, disability, mortality, and higher healthcare costs.^3^ The Asian Working Group for Sarcopenia (AWGS) 2019 guidelines identify three key factors in diagnosing sarcopenia: muscle mass, muscle strength, and physical performance. A diagnosis of sarcopenia is made when low muscle mass is present alongside either reduced muscle strength or impaired physical performance. When all three factors are compromised, the condition is classified as severe sarcopenia.^4^

Aging is also linked to a decline in the function of multiple physiological systems, which may contribute to the development of frailty.^5^ Frailty, as described by the International Association of Gerontology and Geriatrics Frailty Consensus, is characterized by decreased strength and poor physiological functioning, predisposing individuals to greater dependency and increased risk of death.^6^ While there is literature to establish the theoretical foundations of frailty, challenges still exist around its objective assessment. Several validated tools enable standardized assessment of frailty, with the frailty index and frailty phenotype most frequently used.^7^ Since the current definition of sarcopenia considerably overlaps with that of frailty, it may be considered as a component of frailty. Hence, their clinical management is often similar.

Currently approximately 25% of older adults suffer from type 2 diabetes mellitus (T2DM), a chronic disease associated with serious complications, and these numbers are expected to climb in the coming decades.^8^ With increasing human longevity, the prevalence of functional disability and mobility limitations rises; T2DM is a well-recognized risk factor for these outcomes. Numerous studies have shown positive associations of T2DM with sarcopenia and with frailty.^9^ According to the Korean Sarcopenia Obesity Study (KSOS), sarcopenia prevalence in T2DM patients was more than double compared to controls (15.7% versus 6.9%, respectively).^10^

Thus, screening for sarcopenia and frailty is highly relevant for patients with T2DM, as these conditions may be potentially reversible. Early diagnosis and timely intervention in this high-risk population can help prevent the deterioration of lean muscle mass, thereby improving quality of life. This hospital-based study aimed to assess the prevalence of sarcopenia and frailty in ambulatory elderly patients with T2DM seeking healthcare services at our outpatient department.

## MATERIALS AND METHODS

This hospital-based, cross-sectional, single-group prevalence study was conducted in the Departments of General Medicine, Physiology, and Radiodiagnosis at a public teaching hospital. The study enrolled 100 consecutive participants with the following inclusion criteria: (i) age ≥65 years, ambulatory, and visiting the General Medicine outpatient clinic; (ii) a confirmed diagnosis of T2DM; and (iii) either gender who provided consent. Patients with degenerative or inflammatory arthritis of the lower limbs, disabling cerebrovascular accident, Alzheimer’s disease or other cognitive impairment, as well as those with chronic obstructive pulmonary disease, chronic liver disease, or chronic kidney disease were excluded from the study. The participants were enrolled between January 2020 and June 2022 after obtaining clearance from the Institutional Ethics Committee (ECR/658/Inst/PB/2014/RR-2017).

Socio-demographic data were collected from all participants. In addition, as part of a comprehensive geriatric assessment, data on the presence of comorbidities, polypharmacy, personal habits, social support, and screening for depressive symptoms were also collected. A shorter version of the 5-item Geriatric Depression Scale (GDS-5) developed by Hoyl et al. was used.^11^ The GDS-5 was selected due to its diagnostic accuracy comparable to the widely validated 15-item version, which is often less well accepted by older adults.^11^ A score of ≥2 was considered as positive depression screen.

### Sarcopenia Assessment

Sarcopenia was determined using the AWGS 2019 criteria.^4^ These criteria encompass the assessment of both muscle quantity and quality, thereby considering the estimation of muscle mass, muscle strength, and physical performance to categorize patients as having sarcopenia or severe sarcopenia. Various validated tools for measuring these parameters were used in the current study.

### Muscle Mass Estimation

Skeletal muscle mass can be assessed using several techniques, including magnetic resonance imaging (MRI), computed tomography (CT), dual-energy X-ray absorptiometry (DXA), and bioelectrical impedance analysis (BIA). Since both MRI and CT are costly and less accessible for ambulatory, community-dwelling individuals, this study utilized BIA to measure muscle mass. Whole-body composition was evaluated using a multi-frequency BIA device (MC-180, Tanita Co., Ltd, Tokyo, Japan). Participants fasted overnight, then rested seated for 5 minutes before measurement. They then stood barefoot with their feet symmetrically placed on the foot electrodes and arms extended downward while gripping the hand electrodes, per device instructions. The BIA provided a range of measurements, including fat mass, fat-free mass, appendicular skeletal muscle mass (ASM), trunk muscle mass, protein, minerals, total body water, intracellular water, extracellular water, and visceral fat area. Their skeletal muscle index (SMI) was calculated as ASM divided by height squared (kg/m^2^). According to the AWGS 2019 criteria, low muscle mass—defined by a low SMI—was <7.0 kg/m^2^ for men and <5.7 kg/m^2^ for women.^4^,^12^

### Muscle Strength Estimation

Muscle strength can be assessed using several methods, such as hand grip strength (HGS), knee flexion/extension, and peak expiratory flow. Among these, HGS is the most widely used in studies from East and Southeast Asia and was similarly utilized in this study. Hand grip strength was measured using the Jamar Plus Digital Hand Dynamometer (Jamar®, Patterson Medical, Chicago, IL, USA). Participants sat in a standard chair with the shoulder adducted in a neutral rotation, elbow flexed at 90°, with the forearm resting on a table in a neutral position, as per the guidelines of the American Society of Hand Therapists. Three grip strength measurements were taken from the dominant hand, and the average value was used for analysis.^13^ According to the 2019 AWGS criteria, low muscle strength was defined as a HGS <28 kg for males and <18 kg for females.^4^

### Physical Performance Assessment

Physical performance, a measure of whole-body function related to locomotion, can be assessed using gait speed, the Short Physical Performance Battery (SPPB), and the Timed Up and Go (TUG) test. This study used the SPPB, a validated tool widely used in epidemiological research to evaluate lower limb function. It has three components: standing balance, gait speed, and chair stands. For the balance test, participants were instructed to maintain three progressively challenging positions—side-by-side (feet together), semi-tandem (the heel of one foot placed beside the big toe of the other), and tandem (heel of one foot directly in front of the toes of the other)—for 10 seconds each. Gait speed was measured by timing a 6-meter walk at the participant’s usual pace. In the chair stand test, participants were asked to rise from a standard chair (40 cm high, 30 cm deep) five times with their arms crossed over the chest. The total SPPB score, calculated by summing the results of all three tests, ranges from 0 to 12, with scores ≤9 indicating poor physical performance.^4^,^14^

### Frailty Assessment

In the present study, frailty was assessed using the Fried Frailty Phenotype.^15^ It includes five key components: (i) unintentional weight loss of >4.5 Kg (10 lbs) over the past year, (ii) self-reported exhaustion or fatigue, (iii) low levels of physical activity, typically assessed through standardized questionnaires or activity monitoring, (iv) slowness, evaluated by walking speed over a short distance, and (v) weakness, determined by HGS using a dynamometer. Based on the presence of these criteria, individuals were categorized as robust (none of the criteria), pre-frail (one or two criteria), or frail (three or more criteria).^15^

### Statistical Analysis

Data were recorded using a Microsoft Excel spreadsheet, and statistical analysis was performed using R Studio (R Foundation for Statistical Computing, Vienna, Austria). Associations between categorical variables were assessed using the chi-square test; when expected cell counts were below 5, Fisher’s exact test was applied. A *P*-value of less than 0.05 was considered statistically significant. Percentages were rounded to the nearest tenth, non-significant *P*-values to the nearest hundredth, and significant *P*-values to the nearest thousandth.

## RESULTS

A total of 165 participants were screened for eligibility. Of these, 60 were excluded based on the exclusion criteria, and 5 refused to give their consent. Consequently, 100 ambulatory older patients with T2DM who met the inclusion criteria were enrolled in the study, of whom 52% were men. Sarcopenia was present in 30% (severe in 17%). By frailty status, 59% were pre-frail, 27% frail, and 14% robust. Age distribution and other baseline characteristics are shown in Table 1. For sarcopenia, no demographic or clinical variable was significant (all *P*≥0.10; Table 1). Prevalence was higher in the oldest group (37.5%) than in those aged 65–69 (27.9%), but the difference was not significant (*P*=0.45). It was also higher in men than in women (40.4% versus 18.8%), again not significant (*P*=0.10).

**Table 1:** Sociodemographic Characteristics and their Association with Sarcopenia.

Participants (*N*=100)	No Sarcopenia (*n*=70)	Sarcopenia (*n*=30)	Severe Sarcopenia* (*n*=17)	*P*-Value#
**Age group (years), ** ***n*** ** (%)**				
65–69 (*n*=68)	49 (72.1)	19 (27.9)	10 (14.7)	0.45
70–75 (*n*=24)	16 (66.7)	8 (33.3)	5 (20.8)	
>75 (*n*=8)	5 (62.5)	3 (37.5)	2 (25.0)	

**Sex, ** ***n*** ** (%)**				
Male (*n*=52)	31 (59.6)	21 (40.4)	11 (21.2)	0.10
Female (*n*=48)	39 (81.3)	9 (18.8)	6 (12.5)	

**Residence, ** ***n*** ** (%)**				
Urban (*n*=60)	41 (68.3)	19 (31.7)	11 (18.3)	0.66
Rural (*n*=40)	29 (72.5)	11 (27.5)	6 (15.0)	

**Education level, ** ***n*** ** (%)**				
<High school (*n*=58)	38 (65.5)	20 (34.5)	9 (15.5)	0.28
≥High school (*n*=42)	32 (76.2)	10 (23.8)	8 (19.0)	

**Co-habitation, ** ***n*** ** (%)**				
Living with spouse (*n*=78)	57 (73.1)	21 (26.9)	14 (18.0)	0.29
Living alone (*n*=22)	13 (59.1)	9 (40.9)	3 (13.6)	

**GDS-5 SCORE, ** ***n*** ** (%)**				
≥2 (*n*=57)	42 (73.7)	15 (26.3)	9 (15.8)	0.38
<2 (*n*=43)	28 (65.1)	15 (34.9)	8 (18.6)	

**Medical comorbidities, ** ***n*** ** (%)**				
Hypertension (*n*=62)	47 (75.8)	15 (24.2)	7 (11.3)	0.11
No hypertension (*n*=38)	23 (60.5)	15 (39.5)	10 (26.3)	

CAD (*n*=19)	11 (57.9)	8 (42.1)	5 (26.3)	0.26
No CAD (*n*=81)	59 (72.8)	22 (27.2)	12 (14.8)	

Hypothyroidism (*n*=12)	9 (75.0)	3 (25.0)	2 (16.7)	0.69
No hypothyroidism (*n*=88)	61 (69.3)	27 (30.7)	15 (17.1)	

**Polypharmacy, ** ***n*** ** (%)**				
<5 types/day (*n*=53)	36 (67.9)	17 (32.1)	9 (17.0)	0.63
≥5 types/day (*n*=47)	34 (72.3)	13 (27.7)	8 (17.0)	

**Personal habits, ** ***n*** ** (%)**				

Smoker (*n*=18)	10 (55.6)	8 (44.4)	6 (33.3)	0.58
Non-smoker (*n*=82)	60 (73.2)	22 (26.8)	11 (13.4)	

Drinks alcohol (*n*=33)	25 (75.8)	8 (24.2)	6 (18.2)	0.48
Abstains from alcohol (*n*=67)	45 (67.2)	22 (32.8)	11 (16.4)	

Tobacco user (*n*=9)	5 (55.6)	4 (44.4)	2 (22.2)	0.45
No tobacco use (*n*=91)	65 (71.4)	26 (28.6)	9 (9.9)	

* Severe sarcopenia is a subset of sarcopenia, defined as individuals fulfilling all three criteria (low muscle strength, low muscle mass, and low physical performance).

# *P-*values represent comparisons between non-sarcopenic and all sarcopenic participants (including both non-severe and severe).

CAD, coronary artery disease; GDS-5, 5-item Geriatric Depression Scale.

Based on the AWGS 2019 diagnostic components, low SMI, low HGS, and poor SPPB were present in 31%, 72%, and 53% of participants, respectively (Table 2). When compared by sex, low SMI and low HGS were more frequent in men, whereas poor SPPB scores were more frequent in women (Table 2). On continuous measures, men had higher SMI and HGS, while SPPB scores were similar between sexes. Taken together, Table 2 shows a high burden of low muscle mass, weak grip strength, and poor physical performance in this elderly diabetic population, affecting both sexes and highlighting domains where targeted interventions may be warranted.

**Table 2:** Distribution of Sarcopenia Components in Men versus Women.

Component	Men (*n*=52)	Women (*n*=48)	*P*-value^*^
**SMI (kg/m** **^2^** **)**			
Normal, *n* (%)	31 (59.6)	38 (79.2)	0.06
	
Low, *n* (%)	21 (40.4)	10 (20.8)	

Mean±SD	7.62±1.59	6.46±0.94	

**HGS (kg)**			
Normal, *n* (%)	9 (17.3)	19 (39.6)	0.13
	
Low, *n* (%)	43 (82.7)	29 (60.4)	
	
Mean±SD	23.47±6.79	16.79±4.33	

**SPPB** (0–12)			
Normal, *n* (%)	28 (53.8)	19 (39.6)	0.16
	
Poor, *n* (%)	24 (46.2)	29 (60.4)	
	
Mean±SD	8.15±1.79	7.48±2.20	

* *P*-value compares the distribution of Normal versus Low/Poor between men and women (chi-square test).

Cut-offs: SMI Low <7.0 kg/m^2^ (men), <5.7 kg/m^2^ (women); HGS Low <28 kg (men), <18 kg (women); SPPB Normal >9, Poor ≤9.

Total cohort means: SMI 7.06±1.44 kg/m^2^; HGS 20.26±6.63 kg; SPPB 7.83±2.02.

HGS, handgrip strength; SD, standard deviation; SMI, skeletal muscle index; SPPB, Short Physical Performance Battery.

In contrast to sarcopenia, frailty was significantly associated with age and sex. Frailty increased across age groups (*P*=0.042) with the robust proportion shrinking in older patients, and this was more common in women than in men (*P*=0.003). No significant associations were seen for residence, education, or living arrangement, nor for polypharmacy, depressive symptoms, or common morbidities (Table 3).

**Table 3:** Patient Characteristics by Frailty Status.

Participants (*N*=100)	Robust (*n*=14)	Pre-frail (*n*=59)	Frail (*n*=27)	*P*-Value
**Age, years, ** ***n*** ** (%)**				
65–69 (*n*=68)	10 (14.7)	44 (64.7)	14 (20.6)	0.042
70–75 (*n*=24)	4 (16.7)	13 (54.2)	7 (29.2)	
>75 (*n*=8)	0	2 (25.0)	6 (75.0)	

**Sex, ** ***n*** ** (%)**				
Male (*n*=52)	12 (23.1)	32 (61.5)	8 (15.4)	0.003
Female (*n*=48)	2 (4.2)	27 (56.3)	19 (39.6)	

**Residence, ** ***n*** ** (%)**				
Urban (*n*=60)	8 (13.3)	37 (61.7)	15 (25.0)	0.63
Rural (*n*=40)	6 (15.0)	22 (55.0)	12 (30.0)	

**Education level, ** ***n*** ** (%)**				
<High school (*n*=58)	8 (13.8)	36 (62.1)	14 (24.1)	0.56
≥High school (*n*=42)	6 (14.3)	23 (54.8)	13 (31.0)	

**Co-habitation, ** ***n*** ** (%)**				
Living with spouse (*n*=78)	9 (11.5)	46 (59.0)	23 (29.5)	0.31
Living alone (*n*=22)	5 (22.7)	13 (59.1)	4 (18.2)	

**GDS-5 score, ** ***n*** ** (%)**				
≥2 (*n*=57)	10 (17.5)	32 (56.1)	15 (26.3)	0.93
<2 (*n*=43)	4 (9.3)	27 (62.8)	12 (27.9)	

**Medical comorbidities, ** ***n*** ** (%)**				
Hypertension (*n*=62)	6 (9.7)	40 (64.5)	16 (25.8)	0.50
CAD (*n*=19)	3 (15.8)	11 (57.9)	5 (26.3)	0.31
Hypothyroidism (*n*=12)	1 (8.3)	7 (58.3)	4 (33.3)	0.08

**Polypharmacy, ** ***n*** ** (%)**				
<5 types/day (*n*=53)	8 (15.1)	33 (62.3)	12 (22.6)	0.49
≥5 types/day (*n*=47)	6 (12.8)	26 (55.3)	15 (31.9)	

**Personal habits, ** ***n*** ** (%)**				
Smoker (*n*=18)	5 (27.8)	9 (50.0)	4 (22.2)	0.63
Non-smoker (*n*=82)	9 (11.0)	50 (61.0)	23 (28.0)	

Drinks alcohol (*n*=33)	5 (15.2)	19 (57.6)	9 (27.3)	0.92
Abstains from alcohol (*n*=67)	9 (13.4)	40 (59.7)	18 (26.9)	

Tobacco user (*n*=9)	1 (11.1)	5 (55.6)	3 (33.3)	0.62
No tobacco use (*n*=91)	13 (14.3)	54 (59.3)	24 (26.4)	

CAD, coronary artery disease; GDS-5, 5-item Geriatric Depression Scale; SD, standard deviation.

## DISCUSSION

Aging and its associated outcomes are an inevitable reality; however, the change from a healthy adult to a vulnerable, frail older person needs to be identified early to reduce dependency and death. Aging itself increases the risk of sarcopenia, thereby contributing to functional decline. Sarcopenia, an emerging complication in the geriatric population, has been a constantly evolving entity due to ongoing changes not only in its theoretical definition but also in its practical assessment. A systematic review estimating the prevalence of sarcopenia among community-dwelling older adults reported a prevalence ranging from 9.9% to 40%.^16^ Table 4 summarizes various studies that have examined sarcopenia across different population groups in India.^17^–^20^ The wide variation in estimated sarcopenia prevalence can be attributed to several factors. These include the type of population cohort assessed (community-dwelling, hospitalized, or institutionalized individuals), the operational definitions applied for diagnosis, and the use of non-region-specific normative data and cut-offs, which may lead to inconsistent classification of sarcopenia across populations.

**Table 4:** Studies Assessing Prevalence of Sarcopenia Among the Indian Population.

Reference, first author (year)^ref^	Duration	Study Group	Study Type	Criteria Applied	Results
Rao et al. (2025)^17^	2017–2018	Community-dwelling adults aged 60 and above	Cross-sectional, observational	AWGS 2019	Sarcopenia: 43.6% Severe sarcopenia: 19.4%
Rahman et al. (2021)^18^	2015–2016	Participants, aged 60 years and above, attending the geriatric outpatient clinic	Cross-sectional	EWGSOP 2010	Sarcopenia: 39.2%
Bhat et al. (2024)^19^	2020–2023	All individuals above 40 years of age	Cross-sectional	AWGS 2019	Sarcopenia: 10% Severe sarcopenia: 4.2%
Pal et al. (2020)^20^	2016–2019	Healthy individuals from the community aged 20 years and above	Cross-sectional, observational	EWGSOP 2 criteria	Probable sarcopenia: 14.6% Sarcopenia: 3.2% Severe sarcopenia: 2.3%

AWGS, Asian Working Group for Sarcopenia; EWGSOP, European Working Group on Sarcopenia in Older People.

Type 2 diabetes mellitus is a well-recognized risk factor for functional impairment and mobility limitations and has been consistently linked to both sarcopenia and frailty in numerous studies.^9^ However, the mechanisms underlying the frequent coexistence of diabetes with these conditions are not yet fully understood. Type 2 diabetes mellitus is marked by insulin resistance and disrupted insulin signaling, which can impair protein synthesis while promoting protein breakdown—processes that contribute to the loss of muscle mass.^21^ In our study, the occurrence of sarcopenia among elderly individuals with T2DM was higher than reported in several Asian studies of older adults with and without T2DM. For instance, a Chinese study reported a prevalence of 10.37% among participants with T2DM aged >60 years.^22^ Similarly, a meta-analysis of community-dwelling Asian adults residing in Singapore, Japan, China, and South Korea reported prevalence rates of 15.9% in those with T2DM and 10.8% in non-diabetic individuals.^23^ While Chen et al. primarily used BIA for muscle mass assessment,^22^ Chung et al. conducted a meta-analysis of studies estimating muscle mass using either DXA or BIA.^23^

Several factors may account for the higher prevalence of sarcopenia observed in our current study. Firstly, our study setting differed from that of other studies (single-center, lower-middle-income country with participants ≥65 years in our study versus multicenter, ≥60 years, and upper-middle and high-income settings in others).^24^ The comparatively lower prevalence of sarcopenia in those other studies may be attributed to higher levels of education and better access to healthcare in those higher-income settings.^25^ Secondly, differences might also be due to the use of different assessment tools (e.g. our hydraulic dynamometer and SPPB versus the strain-gauge dynamometers and gait speed alone used by Chen et al.^22^), and diagnostic criteria (AWGS 2019 in our study versus the Chinese researchers’ use of AWGS 2014 and other local criteria). However, our observations were quite similar to a study conducted in Singapore by Fung et al., where the authors showed the prevalence of sarcopenia to be 27.4% among patients with T2DM aged ≥60 years.^26^ This highlights that the clinical burden of T2DM in older adults extends beyond traditional microvascular and macrovascular complications, with sarcopenia now emerging as a significant and underrecognized consequence.^27^

Despite the relatively high occurrence of sarcopenia, we did not observe any statistically significant associations between sarcopenia and key demographic or clinical variables in our dataset. In our analysis, sarcopenia status was not significantly linked to age, sex, living situation (e.g. living alone versus with family), comorbidity burden, depressive symptoms, or polypharmacy. This lack of association was somewhat unexpected given the patterns reported in larger studies. For instance, prior evidence has shown that sarcopenia in T2DM tends to be more prevalent with advancing age in male patients.^28^ A 2021 meta-analysis identified older age and male sex as significant risk factors for sarcopenia in T2DM, along with factors such as poor glycemic control and osteoporosis. In contrast, our smaller cohort did not replicate those associations—likely due, firstly, to the limited sample size and consequent reduced power to detect subtle effects, and secondly, to heterogeneity within the study population, as two-thirds of the participants were in the 65–69 age group, while only one-third were in the 70–75 and >75-year age groups combined. Similarly, the influence of sex on muscle mass and strength may not have been detectable here. Although men often have higher absolute muscle mass biologically, studies have found that they experience a more pronounced age-related decline in muscle mass, especially in those with T2DM.^10^,^29^ Although the mean SMI and sex-specific mean SMI of our patients was above the cut-off, our cohort exhibited low muscle strength and poor physical performance. Similarly, an Indian study reported lower HGS among men and women with T2DM despite normal SMI values.^30^ This highlights that muscle strength and physical performance does not always correlate linearly with muscle mass.^29^,^31^ This discordance may be explained by impaired muscle quality, defined as muscle strength relative to regional muscle mass. The Health, Aging, and Body Composition Study further demonstrated that individuals with T2DM had poorer muscle quality across all four limbs compared to those without T2DM.^31^

Frailty is a prevalent geriatric syndrome associated with an elevated risk of adverse health outcomes, such as falls, disability, hospitalization, and mortality, primarily due to diminished physiological reserves.^5^ Among community-dwelling individuals aged 65 years and above, the average frailty prevalence is approximately 10%, though estimates vary widely—from 4.0% to 59.1%—depending on the diagnostic criteria employed.^32^ A recent meta-analysis focusing on community-dwelling older adults in Asia found the prevalence of frailty to be 14.6%.^33^

In contrast to sarcopenia, frailty in our cohort was significantly associated with two patient characteristics: sex and age. Notably, frailty was significantly more common in women than in men. The male–female health survival paradox may help explain this pattern: although women tend to live longer than men, they experience greater morbidity and disability, contributing to a higher prevalence of frailty in this group.^34^ In one large cross-sectional study of multimorbid elderly patients, female sex was associated with nearly two-fold higher odds of frailty.^35^ Our finding aligns with that trend in older adults with T2DM.

Frailty in our participants was also associated with advancing age—older subgroups had higher frailty prevalence, also consistent with the well-established relationship between age and frailty risk. Aging is a primary driver of frailty in most studies, and our data support that frailty becomes more prevalent in the later decades of life, even within an older T2DM cohort. On the other hand, we did not detect significant links between frailty and other variables such as educational level, living situation, overall comorbidity count, or health behaviors. This means that factors like the number of chronic diseases, presence of complications, or lifestyle indicators (e.g. physical activity levels or smoking status) were not clearly associated with frailty status in our analysis.

This absence of association contrasts with some reports in the literature. For instance, a recent meta-analysis indicated that lower exercise levels and poor glycemic control are associated with higher frailty risk in older adults with diabetes.^36^ Additionally, clinical experience and prior studies suggest that polypharmacy, depressive symptoms, and multimorbidity often contribute to frailty due to cumulative stress on physiological reserves, although we did not observe a statistically significant effect in our sample.

Significant losses in muscle mass, strength, and function, along with frailty, are common in older adults with T2DM, but these issues are often inadequately evaluated in standard medical care. While frailty assessment is more comprehensive, involving both qualitative and quantitative aspects of the aging process, sarcopenia has a more operational and objective definition and is considered part of the frailty spectrum. Sarcopenia is believed to act as a transitional phase in the progression toward frailty in individuals with diabetes. Consequently, incorporating regular evaluations of both conditions into clinical practice is essential to minimize the risk of functional deterioration among elderly patients with T2DM.

### Study Strengths and Limitations

The strengths of the current study include, first, the use of the most recent and region-specific diagnostic criteria, the AWGS 2019 criteria, for assessing the prevalence of sarcopenia. This stands in contrast to several other studies conducted in India and Southeast Asian countries that relied on the European Working Group on Sarcopenia in Older People (EWGSOP) criteria.^37^ Second, the study recruited individuals attending the outpatient department rather than community-dwelling older adults. This population is not only more likely to exhibit sarcopenia, pre-frailty, or frailty, but also represents a setting where the use of validated screening tools is both economically feasible and logistically convenient, compared to conducting large-scale community screening programs. Lastly, despite a relatively small sample size, the study effectively captured the burden of frailty and sarcopenia in this high-risk population.

The study had certain limitations. It did not include non-diabetic elderly individuals as a control group, nor did it account for variables such as duration of diabetes, nutritional intake, activities of daily living, and body mass index, all of which may have influenced the development and prevalence of sarcopenia and frailty.

## CONCLUSION

The current research brings to the fore the substantial occurrence of sarcopenia and frailty amongst elderly patients with type 2 diabetes. In our cohort, nearly one-third had sarcopenia, and one-fourth had frailty, underscoring the high burden of these hidden morbidities. Identifying this vulnerable group through early and routine screening will support optimal allocation and utilization of healthcare resources and aid in the restoration of muscle strength and function.

## Abbreviations

ASM: appendicular skeletal muscle mass
AWGS: Asian Working Group for Sarcopenia
BIA: bioelectrical impedance analysis
CT: computed tomography
DXA: dual-energy X-ray absorptiometry
EWGSOP: European Working Group on Sarcopenia in Older People
GDS-5: 5-item Geriatric Depression Scale
HGS: hand grip strength
MRI: magnetic resonance imaging
SMI: skeletal muscle index
SPPB: Short Physical Performance Battery
T2DM: type 2 diabetes mellitus.
